# Why is it difficult to implement e-health initiatives? A qualitative study

**DOI:** 10.1186/1748-5908-6-6

**Published:** 2011-01-19

**Authors:** Elizabeth Murray, Joanne Burns, Carl May, Tracy Finch, Catherine O'Donnell, Paul Wallace, Frances Mair

**Affiliations:** 1 e-Health Unit, Department of Primary Care and Population Health, University College London, Royal Free Campus, Rowland Hill Street, London NW3 2 PF, UK; 2 Primary Care Research Network for Greater London, London South Bank University, 103 Borough Road, London SE1 0AA, UK; 3 Faculty of Health Sciences, University of Southampton, Southampton SO17 1BJ, UK; 4 Institute of Health and Society, University of Newcastle, UK; 5 Academic Unit of General Practice and Primary Care, Centre for Population and Health Sciences, College of Medical, Veterinary and Life Sciences, University of Glasgow, 1 Horslethill Road, Glasgow G12 9LX, UK

## Abstract

**Background:**

The use of information and communication technologies in healthcare is seen as essential for high quality and cost-effective healthcare. However, implementation of e-health initiatives has often been problematic, with many failing to demonstrate predicted benefits. This study aimed to explore and understand the experiences of implementers -- the senior managers and other staff charged with implementing e-health initiatives and their assessment of factors which promote or inhibit the successful implementation, embedding, and integration of e-health initiatives.

**Methods:**

We used a case study methodology, using semi-structured interviews with implementers for data collection. Case studies were selected to provide a range of healthcare contexts (primary, secondary, community care), e-health initiatives, and degrees of normalization. The initiatives studied were Picture Archiving and Communication System (PACS) in secondary care, a Community Nurse Information System (CNIS) in community care, and Choose and Book (C&B) across the primary-secondary care interface. Implementers were selected to provide a range of seniority, including chief executive officers, middle managers, and staff with 'on the ground' experience. Interview data were analyzed using a framework derived from Normalization Process Theory (NPT).

**Results:**

Twenty-three interviews were completed across the three case studies. There were wide differences in experiences of implementation and embedding across these case studies; these differences were well explained by collective action components of NPT. New technology was most likely to 'normalize' where implementers perceived that it had a positive impact on interactions between professionals and patients and between different professional groups, and fit well with the organisational goals and skill sets of existing staff. However, where implementers perceived problems in one or more of these areas, they also perceived a lower level of normalization.

**Conclusions:**

Implementers had rich understandings of barriers and facilitators to successful implementation of e-health initiatives, and their views should continue to be sought in future research. NPT can be used to explain observed variations in implementation processes, and may be useful in drawing planners' attention to potential problems with a view to addressing them during implementation planning.

## Background

The challenges facing healthcare systems in the twenty-first century have been well described: an aging population; increasing prevalence of long-term conditions; improving health technologies leading to better survival; and rising expectations of healthcare all combine to put ever increasing pressure on available healthcare resources [1]. Although each country is pursuing individual solutions to these challenges, some common approaches are clearly apparent, including the use of information and communication technology (ICT) [2]. The use of ICT is expected to lead to improvements in healthcare quality (*e.g.*, through better communication) and efficiency (*e.g.*, through reduced duplication of investigations) [3]. Australia, New Zealand, and the UK have been at the forefront of attempts to embed ICT into routine healthcare [4], with the UK investing £12.4 billion over 10 years [5]. However, despite political commitment and substantial investment, there has been significant variability in the success of different e-health implementations across the British National Health Service (NHS) [6]. Many projects have been subject to considerable delay, increasing budget deficits, and in some cases, severely negative impacts on the quality and effectiveness of care [7,8].

Difficulties in e-health implementation are an international phenomenon, with similar problems being widely reported [9-12]. This work has taken many forms and, importantly, it has raised questions about what 'successful' implementation actually means. For example, de Bont and Bal [13] have described how a telemedicine service met organizational criteria for 'success' and yet failed to normalize in practice. Despite this critical conceptual problem, much research has focused on issues of efficacy or effectiveness, with trials addressing the 'can it work/does it work?' questions [2,3]. How new systems are 'implemented' remains a problem, and an important theme in much recent work has been the problem of 'resistance' or refractory behaviours of professionals -- and the assumption that their 'attitudes' to e-health are the root problem [14]. Studies exploring the views of senior staff charged with implementing an e-health innovation are rare [15]. This is surprising, because these people (henceforth referred to as 'implementers'), with their direct experience of planning and managing implementations, are likely to have useful perspectives on the factors contributing to the success or failure of new systems, which might contribute to bridging the gap between research and its wider implementation into practice [16,17]

Although there is a considerable body of work on factors promoting successful implementation in healthcare [18,19], implementation research within healthcare has been described as a 'relatively young science' [20]. This is reflected in vigorous debates about how to understand implementation processes and about the theoretical tools that can be used to do this [21]. These offer us generalisable frameworks that can apply across differing settings and individuals; the opportunity for incremental accumulation of knowledge; and an explicit framework for analysis [21]. There are a number of theoretical frameworks that have been applied to studies of technological change in healthcare and informatics, and important contributions have been made to understanding the role of attitudes [22], and social transmission of innovations between [23] or interactions within [24,25] actor-networks. More recently, Greenhalgh *et al. *have offered a high level and abstract theorization of ICT programmes from the perspective of Structuration Theory [26].

Like de Bont and Bal [13], Berg [24], and Greenhalgh and Stones [26], our study falls within the general frame of science and technology studies [27]. However, we were interested in taking a social action approach to implementation, rather than focusing on socio-technical relations or higher-level theories of structuration. We wanted to understand the work that implementers did, and our approach was informed by the analysis of collective action, a core construct of Normalization Process Theory (NPT) [28], which we used to provide a general framework for this study. In particular, we focused on those of its components [29] that support the analysis of enacting implementation and other social processes. NPT focuses on the work that individuals and groups have to do for a new technology or practice to become embedded and sustained in routine practice.

**Figure 1:**
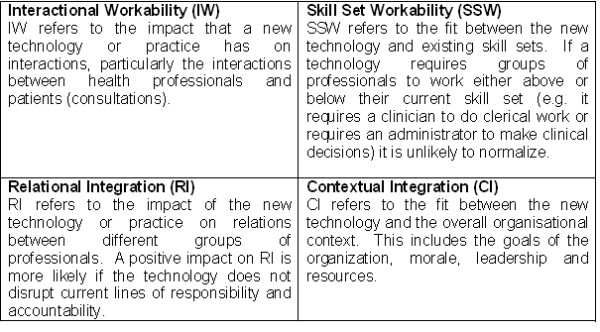
**Constructs of the collective action component of normalization process theory**.

We were interested in exploring the application of four of NPTs concepts: interactional workability (IW); relational integration (RI); skill set workability (SSW); and contextual integration (CI) (Figure 1). IW refers to the impact that a new technology or practice has on interactions, particularly the interactions between health professionals and patients (consultations). RI refers to the impact of the new technology or practice on relations between different groups of professionals, and the degree to which it promotes trust, accountability, and responsibility in inter-professional relationships. SSW refers to the fit between the new technology and existing skill sets. An example of poor SSW would be a technology that required clinicians to do clerical work, or conversely, required administrative staff to take clinical decisions. CI, which refers to the fit between the new technology and overall organisational context, including organisational goals, morale, leadership, and distribution of resources.

The assumption that informed our analysis was that technologies that are understood by their users to have a positive impact on consultations (IW), inter-professional relationships (RI), and which fit well with existing skill sets (SSW) and organisational context (CI) are more likely to normalize than those with a negative impact or poor fit [30].

This study had two aims: first, to determine implementers' views of factors which promote or inhibit successful normalization (implementation, embedding, and integration) of e-health innovations; and secondly, to explore whether the collective action components of Normalization Process Theory (NPT) provided an adequate explanation for different perceived degrees of normalization. Although NPT was derived from a large body of empirical work, at the time this study was designed (2006), there were relatively few studies which had attempted to test NPT's power as an explanatory model across a range of technologies [31-33]. We adopted a case study methodology as the most effective way of addressing these two aims because case study methods are appropriate for studying complex systems which are in a state of flux [34] and for exploring why and how particular outcomes occurred, rather than simply describing what happened [35]. Case study methods are distinguished by their in-depth focus on a relatively small number of units or 'cases' [36], and benefit from prior development of theoretical propositions to guide data collection and analysis [37].

## Methods

### Design

We report case studies of three e-health innovations. Data were collected using semi-structured interviews with implementers and analyzed using the Normalization Process Model.

### Setting

Our theoretical framework, as well as previous research conducted by members of the team [38,39], led us to postulate that the characteristics most likely to influence the success or failure of an implementation were the clinical context (primary, secondary, or community care) and the nature of the e-health technology [29]. In addition, we wished to ensure that the implementation was recent enough to remain alive in respondent's memories, while sufficiently established to allow for assessment of the extent to which the initiative had become embedded and integrated into routine practice (normalized). These criteria led to the selection of three cases (Table 1). In each case, the implementation had occurred between 2004 to 2006, with data collection undertaken 2007 to 2008.

**Table 1:** Summary of Case Study characteristics

	Case Study		
	Choose and Book	Picture Archiving and Communication System	Community Nurse Information System
Health care setting	Primary/Secondary care interface	Secondary care	Community care
Aim of technology	Allow patients to book first outpatient appointment at hospital of choice	Digitise x-rays and other images so they can be stored and viewed electronically	Electronic record system that also allows patient registration, clinic and visit scheduling and access to clinical algorithms.
Professionals affected by technology	Primary care: GPs, administrative staff. Secondary care: Consultants, outpatient administrative staff	Doctors, radiologists, radiography administrative staff	Community nurses

Case study one (CS1) was the implementation of the Choose and Book (C&B) system in a hospital trust serving an inner city population in a large metropolitan area in England and the lead Primary Care Trust providing referrals to that hospital. C&B was a national electronic service that provided patients with the opportunity to choose which hospital their general practitioner (GP) referred them to for a particular problem, and to book the time and date of their first appointment. C&B was a flagship project for the multi-billion pound programme for improving use of information technology in the English NHS, known as Connecting for Health [40]. Implementation involved three main stakeholders: the hospital receiving referrals, the Primary Care Trust (PCT) commissioning out-patient appointments, and the GPs making referrals.

Case Study two (CS2) was the implementation of the Picture Archive and Communication System (PACS) in one acute hospital trust, which included several hospitals at different sites, located in a largely rural area of England. PACS was a system for digitizing images, such as X-rays, scans, or photographs. The digitized images could be stored online, and accessed simultaneously from different locations.

Case Study three (CS3) was the implementation of a Community Nursing Information System (CNIS) for district nurses in an urban area in Scotland. The CNIS consisted of hand-held wireless enabled Personal Digital Assistant devices (iPAQs). District Nurses could use them to record clinical assessment information while out in the community, and download the information to the central server once back at base. The system also included some decision support in the form of standardized assessment tools with associated care algorithms. The system had originally been intended to form a single shared assessment that could be shared between district nurses and social services; however, social services had been unable to pursue their side of the implementation and so this function had not become available by the time of data collection.

### Participants

**Figure 2:**
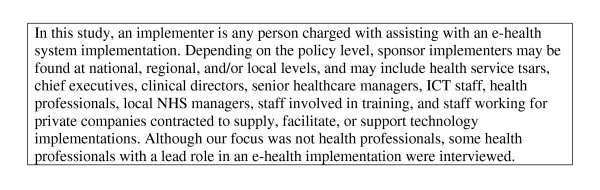
**Definition of implementers**.

Participants were staff with responsibility for planning and/or executing an e-health initiative ('implementers' as defined in Figure 2). We purposively recruited a maximum variety sample, aiming to include senior Department of Health or Connecting for Health staff with responsibility for a number of e-health projects across multiple organizations, senior staff from within the Trust or Health Board with lead responsibility for implementing a number of e-health systems within their organization (such as chief executive officers), and middle management with day-to-day responsibility for the implementation under study. Recruitment within each case study continued until we reached saturation, *i.e.*, until no new data were emerging from subsequent interviews. Based on previous experience, we estimated that up to ten interviews per case study would be needed [38].

### Data collection

Semi-structured interviews were used to determine not only 'what happened' but also participants' explanations of 'why it happened' in that way. Interviewees were asked for a description of the e-health implementation process from their perspective, their views about factors which had promoted or impeded implementation and their assessment of how normalized (embedded into routine care) the e-health initiative had become. Interviews were tape-recorded and transcribed verbatim, with the interviewer keeping additional field notes.

### Data analysis

Data were analyzed using the framework method proposed by Ritchie and Spencer [41] according to four components of the collective action construct of NPT (May 2006): IW, RI, SSW, and CI (Figure 1). Data were coded to the four constructs and overall degree of normalization.

Initial interviews were coded by the interviewer (JB) and chief investigator (EM) in order to develop a coding framework. This framework was then tested and refined at a two-day multidisciplinary data analysis clinic involving all authors. The revised coding frame was reapplied to the previously coded interviews and all subsequent interviews by three authors independently (JB, EM, CM). There were no significant disagreements in applying the coding framework.

Data are presented in the text with each quotation followed by case study number and role of interviewee. Where quotes include remarks by the interviewer, the interviewer is denoted by 'I' and the participant by 'P.'

## Results

Twenty-three interviews were undertaken: ten for CS1, five for CS2, and eight for CS3. Our intended sampling frame was achieved, with interviewees including regional leads for the cluster (CS2) or local service provider (CS1), Chief executives for the trust or health board for all three case studies, and clinical or IT leads and a range of middle management with 'on the ground' responsibilities (Table 2). Data saturation was achieved quickly in the two case studies (CS2 and 3), which were located in a single context, but took longer in C&B, where there were very different perspectives emerging from the three different groups of stakeholders in the hospital, the primary care trust, and individual general practices.

**Table 2:** Roles of Interviewees

Case Study	Choose and Book (CS 1)	PACS (CS 2)	CNIS (CS 3)
Regional Level	Lead for Local Service Provider	Regional Implementation Director for Cluster	
Chief Executive	CEO of Trust	CEO of Trust	Managing Director of provider company; General Manager of Health Board
Senior Management	Clinical Lead for Hospital Trust	Clinical Lead for Hospital Trust	IT Manager Health Board; Clinical Services Manager
Middle Management or "on the ground"	GP and clinical lead in PCT; Consultant; Practice Manager; Project Manager for Hospital Trust; Outpatient Manager; Primary Care Director for Hospital Trust	Radiology Manager; IT Manager	Lead Project Nurse; IT training manager Health Board; Senior Nurses x 2

### Assessments of normalization

For each case study, we explored interviewee perspectives of the degree to which the e-health innovation had become normalized. Data were triangulated across the different interviewee perspectives. The three case studies demonstrated a wide range of normalization (Table 3). For example, CS2 (PACS) had completely normalized and was totally embedded into routine practice:

**Table 3:** Summary of factors affecting normalization of study technologies

Case Study	C & B (hospital)	C & B (primary care)	PACS	CNIS
Degree of normalization	✓✓✓	✗/✓	✓✓✓	✓
Interactional Workability (impact on consultations)	✗	✗✗✗	✓✓✓	✓
Relational Integration (impact on inter-professional relationships)	✗	✗	✓✓	✗/✓
Skill Set Workability (fit with existing skill sets)	✓	✗✗	✓	✗✗✗
Contextual Integration (fit with organizational context)	✓✓✓	✗/✓	✓✓✓	✓

'It's just taken for granted that you come in and you use PACS and that's how your images are that's it... Just normal practice now.' --CS2 IT training manager

In contrast, CS3 (CNIS) had at best, only partially normalized, and provided a good example of the difference between adoption and normalization. Although some 80% of the district nurses were using it, many teams were still running dual systems (old paper-based and new electronic), and it was evident that not all nurses felt comfortable using it, with the hand-held devices still seen as new or strange:

'I think it's fair to say it's not integrated into normal routines very much at all in my area, but the previous area that they were in before, they, I mean, I understand that they have been started last May, and they're only 80% on the system.' --CS3 senior nurse

'It's a new gadget to show off amongst their friends and stuff like that.' --CS3 IT trainer

The picture in CS1 (C&B) was more complex. It appeared that there had been a high degree of normalization in the hospital, with references to it as 'a way of life here' (CS1 hospital chief executive officer) or 'completely embedded in standard operational workings' (CS1: project manager for C&B in the hospital). In primary care, there was variable (and often low) normalization with certain practices contributing the bulk of the electronic referrals:

'Yeah, well most GPs don't use it!' --CS1 hospital chief executive officer

Even in those practices that were high users of C&B, it was considered problematic, and had not become part of routine practice:

'Right you are saying within my 10-minute slot and you have said Choose and Book will take a couple of minutes -- it doesn't -- what, even two and a half years on it takes at least four and is not even working properly today. So it took me 10 minutes to do one this morning.' --CS1 GP early adopter

This variability in perceived normalization was further analyzed using NPT as an explanatory framework (Table 3). Where implementers perceived good levels of CI, IW, RI, and SSW, high levels of normalization had occurred. However, where implementers perceived problems in one or more of these areas, the level of normalization was lower.

### Interactional workability

Data were considered to refer to IW if they reported the impact of the new technology on health professional -- patient interactions or consultations. PACS was perceived as having a very positive impact on doctor-patient relationships on two grounds. The first was that images were always available when needed, allowing clinicians to make decisions in a timely manner:

'The biggest advantage is in having images available all the time to everyone. So as soon as I take a picture of you, somebody can see it. In fact, everybody can see it. So where, if you were come into A and E and you've broken an arm and you have to be referred to the orthopaedic surgeons, there is no backwards and forwards of one piece of film following you around or not as the case may be. The fact that you have a picture that any doctor can see, the orthopaedic surgeon can see; it can be in the theatre if you get up there in 10 minutes time. It can be on the ward if you are admitted to the ward, it can be in the department for specialist, um, review of it and report being done -- all at the same time.' --CS2 radiology manager

Second, doctors liked being able to show patients their images, and found this easier to do with PACS than with film:

'you did get good doctors saying 'it's so nice being able to point things, and rotate things, and show things more easily,' because you can magnify and things like that I suppose, so you can do that sort of thing, and share that with the patient.' --CS2 IT training manager

The data suggested that the CNIS had a positive impact on IW. The iPAQ devices were cheap, robust and portable, allowing nurses to feel comfortable carrying them around as they visited patients, and hence providing access to the patient record during home visits:

'You've seen how streamlined they are quite you know petite. You can put them in your pocket.' --CS3 IT trainer

'[Before the CNIS] if you needed information about someone whose condition had deteriorated, perhaps on a Friday afternoon, you then had to write a different set of documentation and drive it to the place that the patient needed to be seen, otherwise there was no way of getting the information to them.' --CS 3; Clinical Services Manager

In contrast, C&B had a negative effect on IW in general practice, with interviewees commenting adversely on the time required to make a C&B referral and the negative impact this had on patient consultations. C&B had little impact on IW in hospital, except where the system allowed patients to be booked into the wrong clinic, which led to unsatisfactory consultations.

### Relational integration

Data were coded to RI if they referred to the impact of the new technology on relationships between groups of professionals.

PACS was reported as promoting communication and trust between different professional groups because it enabled multiple users to view the same image from different locations. This was felt to have improved working relations between for example, orthopaedic surgeons and radiologists, or within multidisciplinary team meetings for planning complex cancer care for individual patients:

'Yes and I think its aiding clinicians to have a better conversation if you put it in the cancer or renal unit ...the multidisciplinary team meeting.... I can remember, my senior pathologist has just retired and she said sitting in some of these meetings now and you've got the pathology there and you've got the images there and she said the quality of the clinical conversation that's going on around what's best for an individual patient and their circumstances has moved on and is a higher quality clinical discussion which I would then argue must lead to better treatment planning and clinical decision making and therefore must lead onto better outcomes for patients.' --CS2 chief executive officer

'And I think, particularly with the interaction between say one of the clinicians and one of the radiologists, that's improved because the consultant outside knows that the consultant radiologist inside has access to those images -- and has probably already seen them, probably already done a report -- so what they are doing is they are starting off from another point. In the old days, if a CT scan was done and it went to the ward, the consultant on the ward would have to pick it up and bring it down to the radiologist and that would be the first time the radiologist was seeing it. Because it had never come down from the ward before. Whereas now, he rings him up and say -- 'you've seen so-and-so, and said so-and-so -- what about this little bit over there?' And then he looks up and ... Or they still come down to the department to talk because they like the interaction, but it is not the first time the radiologist is seeing that scan.' CS2 radiology manager

The CNIS had been intended to have a positive impact on inter-professional relationships because it was originally intended to form the basis for a joint record held by both social services and community nurses. However, problems within social services led to extensive delay, and at the time of data collection, social services were not using the system, preventing any positive impact of the system on RI.

The impact of C&B on relations between professional groups was most marked for the relations between hospital consultants and GPs, with both groups regretting the loss of personal contact between referring doctor and specialist (negative impact on RI):

'I think one of the points about Choose and Book was to basically - is part of a systematic disenfranchisement of clinicians basically - so that we now refer to a generic gastroenterologist or a generic chest physician.' --CS1 GP early adopter

'I think it is all a bit more distant. Because it used to be the GPs referred to their main buddies. And they can't really do so much anymore. What we hope is we substituted for that the confidence that they patients will be seen the first time by someone who can deal with the problem.' --CS1 consultant and clinical lead for C&B in hospital

### Skill set workability

Data were coded to SSW if they referred to the fit between the new technology and existing skill sets, or efforts made to teach the requisite skills to users.

In many ways PACS fit well with existing skill sets. It was seen as relatively intuitive to use, and intensive efforts were put into training clinical staff before implementation:

'... and basically there were a number of sessions set up by our training department with five or six web browsing terminals, and they just went in and they [clinical staff] were shown how to get into their patient; they were shown how to pick an image, and how to adjust and image and read a report. I think we probably got about 60% of the clinical staff in the trust trained before go-live.

I: Before go-live. Oh fantastic.

P: Which was bad. And the other 40% very quickly learnt afterwards.' --CS2 radiology manager

Some clinicians were used to nurses displaying images for them, and were initially reluctant to have to take on that task themselves. However, the advantages of PACS swiftly won them over:

'And the orthopaedic surgeon said 'What happens when I go on the ward and the nurse can't get the image up on the screen?' 'The nurse can't get the image up on the screen -- you're going to!' And off he went, mumbling that he didn't want PACS introduced until he retired. He's now on that DVD that was done as a champion of it.' --CS2 radiology manager

Ease of use was seen as essential for the CNIS, where the nurses started from a low level of IT literacy. Many were alarmed that poor IT skills could jeopardize their future employment:

'It's basically nurses who don't even have a computer in their own homes and they haven't actually come across this sort of technology and they're having to face it at work and sometimes you get that sort of nervous reaction that they maybe might feel a bit inadequate in the sense that that oh this is really daunting. I've never used a computer system before. Will this mean I'll be out of a job?' --CS3 IT trainer

Trainers had to spend a great deal of time on one-to-one training and emotional reassurance:

'I must say, to be honest, they we do hold their hand quite a lot and we've probably spoilt them in a sense that we tend to go out to the health centres and actually do the training rather than tell them to come out to an unfamiliar environment.' --CS3 IT trainer

C&B fit well with the skill sets in hospital, where administrative and IT staff tended to deal with it. In general practice, C&B had a poor level of SSW because GPs were expected to make the C&B referral within a consultation. They perceived this as a clerical function that was a poor use of their clinical skill:

'I think the doctors would say that they are doing a bit more with Choose and Book administration than they used to. They are not happy about that. Really. And that is why that brings out the worst headlines in the comics - 'I am not a travel agent' sort of thing...' --CS1 GP early adopter

### Contextual integration

Data were coded as pertaining to CI if they reported on the fit between the technology and the overall organizational context, including organizational goals, the quality of leadership within the organization, resources allocated to the implementation, and overall morale.

PACS was perceived as a way of meeting several organizational goals, including national targets for shorter waiting times for investigations, increased efficiency within the hospital, and the chief executive officer's personal goal of encouraging clinical engagement with IT. PACS helped the organization achieve their goals by eliminating the problem of x-ray films that had been 'lost' or were unavailable at the time and place they were needed:

'they were never in the right place at the right time. Well, never is too strong a word, but I think there were times when we were running up to about 20% lost films. And what I mean by 'lost films' is just not being in the right place at the right time.' --CS2 radiology manager.

This had considerable knock-on costs in terms of repeat X-rays, delays to consultations or treatments, and staff time in looking for films. PACS eliminated this inefficiency: 'through PACS we become more efficient, more productive' --CS2 consultant radiologist

The chief executive officer was very committed to introducing PACS and provided strong leadership for the implementation process, ensuring that sufficient resources, including time, senior staff and funds were available for the implementation to go well and complete on time:

'Well I drove it, I chaired the project board...It's about change and the way we do things, changing the culture. So I chaired the project board and brought the relevant people, so the lead radiologist who was my key clinical champion was there. My head of IT was there. There were other people involved and in a sense we do everything here by project management methodology. That's the way we make sure we deliver things.' --CS2 chief executive officer

The data from CS3 (CNIS) demonstrated both positive and negative features about CI. On the positive side, the system was seen as a way of achieving the policy goal of sharing assessment information between community nursing and social services. This enabled funds to be identified and targeted on this implementation, while also achieving a long-term goal of engaging a professional group that had little experience of IT:

'This was a, a group of staff who had no access to electronic record-keeping at all. And there had been a series of efforts to do this over the years, and over the previous decade, all of which had failed to... failed over in... to be rolled out... But also -- and this is the other driver was -- that as the rest of the world, all the other service providers that they were engaging with, were increasingly becoming... conducting their business through, through the electronic medium, if they had... if at the very minimum, if you get them onto a platform, if I use that expression, to get them onto something which would enable a, a transfer maybe at some future date, to, to another potential system, depending on what their various service partners may, may develop, because if you're simply not on anything, then it becomes quite difficult to, to be part of an information technology strategy for, for the wider sector... It would introduce them to - as individuals, as professionals - to this world of electronic record-keeping and information sharing, which they just simply had no experience of.' --CS3 director, community health and care partnership

On the negative side, there had been significant organizational change locally, which had absorbed staff time and energy, distracting them from the e-health implementation:

'It's a huge piece of change, re-organizational change at the time we were trying to introduce this, coupled with the Agenda for Change, means we'd three big things that did create issues, and we just had to kind of manage our way around it.' --CS3 joint services manager

'A lot of the nurses just feel it's been one constant change after another.' --CS3 lead project nurse

Possibly related to this organizational change was a perceived problem with leadership, including the disbanding of the dedicated implementation group after the first year and inadequate allocation of resources for training and support, leaving nurses without the input needed to build their confidence and expertise with the system:

'Um, I think a couple of years ago, there was a steering group set up to move this forward. And there was also a reference group set up to look at what should be on the system. Um, because of organizational change, more than anything, I think we've lost the implementation group... I think, really, what's been happening in [city] is that some training has been given to nursing staff, but there's been no follow-up within that area to make sure it's happening.' --CS3 senior nurse

'not having help out of hours. I'm not sure if that's resolved yet; they hadn't resolved it when I moved in 2007 because there was no helpdesk out of hours. They would train the staff and support them but they only worked nine till five, Monday to Friday.' --CS3 clinical services manager

CI of C&B varied according to context. The hospital we studied was in competition with 3 or 4 others located within a few miles, including highly regarded teaching hospitals. The overall number of referrals from primary to secondary care was decreasing, and the study hospital could only survive financially if it could attract an increasing proportion of a decreasing pool of referrals. C and B became a central part of this hospital's business plan to maintain inward referrals and hence overall financial viability:

'So I wanted to make it so easy to book an appointment in this hospital that people would start to use this hospital for booking.' --CS1 hospital chief executive officer

Awareness of this overwhelming importance of C&B to the organization's survival plan had permeated every level of management, leading to considerable investment of energy and resource into the implementation:

'we had very strong executive leadership so it was always top of the priority. We had quite a strict project methodology in terms of the meeting structures that we had. And we had a project board that met consistently and was chaired by chief execs.' --CS1 project manager for C&B in the hospital

During the study period, however, C&B bore little relationship to the goals of the Primary Care Trust or the general practices, apart from an awareness of the government promotion of policies aimed at improving patient choice. Some individual general practices saw the electronic booking component of C&B as a way of cutting down on administrative time spent chasing appointments in secondary care for their patients, but this advantage was often offset by the amount of administrative time taken sorting out problems caused by C&B:

'because we felt there would be real advantages to it and it would hopefully streamline the process of referring patients to hospital and from the whole starting point here through to when the patient was actually seen at the other end. That was what we initially thought.' --CS1 practice manager

## Discussion

Senior staff with responsibility for implementing new e-health technologies in the NHS had clear views about factors that promoted or inhibited perceived normalization of these technologies from their perspective of being involved in service implementations. NPT -- with its emphasis on the degree to which a new technology fits with professional-patient interactions, relationships between staff groups, existing skill sets, and organisational context -- provided a good explanation for the observed variability in normalization of three contrasting technologies in different contexts.

Strengths of this study include the use of case study methodology with case studies selected to include a range of healthcare contexts and types of e-health initiatives. Identifying 'implementers,' a previously under-studied group, proved straightforward, and they did provide data from a perspective that differed to clinicians. The multidisciplinary nature of the research team, the convening of a data clinic to refine the coding framework, and the independent coding by three authors all added to the reflexivity and rigour of the research [42]. Weaknesses include the relatively small number of case studies due to resource constraints and the low number of interviews. A wider range of case studies would have been useful in considering the common features of 'successful' implementation. At the time that this study was performed, the collective action components of NPT were those that were best developed and had survived robust processes of construct validation. We therefore focused analysis through that lens. However, as the study continued other constructs of NPT also reached construct validation stage [43]. We do not think, however, that more interviews per case study would have materially strengthened our findings. It could be argued that the study is weakened by our reliance on interview data, which must of necessity present subjective interpretations of activity and observed phenomena. Observation is the 'gold standard' of socio-technical studies (STS) research but in practice is hard to accomplish in studies like this without large numbers of fieldworkers and privileged access to often contentious and complex settings. We had to do the best we could with resources and ethics committee permissions available to us. The latter was an important restriction on our work, since it was a condition of ethical committee approval that all respondents in this study were given 24 hours to consider and make informed consent before we interviewed them. Documents would have been useful to us, but much of what we were interested in did not reside in documents but rather in knowledge in transit (emails, telephone conversations, *ad hoc *conversations, and meetings) that are hardly ever available to the researcher. Our ethics committee approval made it impossible for us to pursue *ad hoc *conversations; therefore, interviews were the only window onto events that happened far from the researcher's gaze. We note that they seem to be more frequently and intensively used in STS studies generally, perhaps reflecting the increasing complexity of fieldwork arrangements as STS work like ours shifts into the more distributed social spaces of 'whole systems.'

Our qualitative data on normalization of two of the case studies fits with published quantitative data. The problems with C&B that were occurring at the time of our data collection are well documented, with just 63 referrals made using C&B in the first year [44], and Primary Care Trusts only halfway to the C&B target in 2007 [45]. A questionnaire study found that the majority of GPs were not in favour of C&B, citing problems with time constraints and the inflexibility of the system [46], reflecting our finding that poor IW impeded normalization in primary care. In contrast, the literature on PACS suggests that this has been widely adopted internationally [47], accompanied by marked improvements in workflow [48], reporting times, productivity [49], and reduced requests for repeat x-rays [50]. An early interview study in one hospital reported user preference for PACS over traditional films because of improved ability to share images between clinicians (RI), faster reporting times (CI), and potential benefit for patients (IW) [51].

## Conclusions

Two substantive conclusions can be drawn from this work. The first is that there is considerable value in seeking and reporting the views of implementers. Their perspective has been under-studied to date, and yet their experience and expertise gained through direct involvement in planning and managing implementations provides messages of generalisable significance. Second, our findings suggest that NPT provides a useful framework for understanding the processes that affect the implementation, embedding, and integration of new technologies into healthcare systems. Initiatives that have a good fit with existing organizational goals and staff skill sets, as well as a positive impact on patient-professional interactions and relationships between professional groups are likely to normalize. Difficulties in any one area should alert policy makers and senior managers to potential problems that may require pre-emptive action, while difficulties across all four areas may require reconsideration. Further work on the predictive value of NPT is warranted.

## Competing interests

CRM led on developing NPT, and all authors have made important contributions to its development.

## Authors' contributions

All authors have made substantial contributions to the conception and design of the study, have been involved in drafting and revising the manuscript and have approved the final version. JB collected the data for case studies one and two; EM, JB and CM coded the data. EM is the guarantor of the paper. FM was PI on the grant that funded this work.
